# Effects of PACAP Deficiency on Immune Dysfunction and Peyer’s Patch Integrity in Adult Mice

**DOI:** 10.3390/ijms251910676

**Published:** 2024-10-03

**Authors:** Jason Sparks, Matyas Meggyes, Lilla Makszin, Viktoria Jehn, Hedvig Lugosi, Dora Reglodi, Laszlo Szereday

**Affiliations:** 1 Department of Anatomy, HUN-REN-PTE PACAP Research Team, Centre for Neuroscience, Medical School, University of Pecs, 7624 Pecs, Hungary; jason.sparks@aok.pte.hu (J.S.); jehn.viki00@gmail.com (V.J.); hedviglugosi@gmail.com (H.L.); 2 Department of Medical Microbiology and Immunology, Medical School, University of Pecs, 7624 Pecs, Hungary; meggyes.matyas@pte.hu (M.M.); szereday.laszlo@pte.hu (L.S.); 3 Janos Szentagothai Research Center, 7624 Pecs, Hungary; lilla.makszin@aok.pte.hu; 4 Institute of Bioanalysis, Medical School, University of Pecs, 7624 Pecs, Hungary

**Keywords:** PACAP, Peyer’s patches, galactin-9, TIM-3, PD-1, PD-L1

## Abstract

PACAP (pituitary adenylate cyclase activating polypeptide) is a widespread neuropeptide with cytoprotective and anti-inflammatory effects. It plays a role in innate and adaptive immunity, but data are limited about gut-associated lymphoid tissue. We aimed to reveal differences in Peyer’s patches between wild-type (WT) and PACAP-deficient (KO) mice. Peyer’s patch morphology from young (3-months-old) and aging (12–15-months-old) mice was examined, along with flow cytometry to assess immune cell populations, expression of checkpoint molecules (PD-1, PD-L1, TIM-3, Gal-9) and functional markers (CD69, granzyme B, perforin) in CD3+, CD4+, and CD8+ T cells. We found slight differences between aging, but not in young, WT, and KO mice. In WT mice, aging reduced CD8+ T cell numbers frequency and altered checkpoint molecule expression (higher TIM-3, granzyme B; lower Gal-9, CD69). CD4+ T cell frequency was higher with similar checkpoint alterations, indicating a regulatory shift. In PACAP KO mice, aging did not change cell population frequencies but led to higher TIM-3, granzyme B and lower PD-1, PD-L1, Gal-9, and CD69 expression in CD4+ and CD8+ T cells, with reduced overall T cell activity. Thus, PACAP deficiency impacts immune dysfunction by altering checkpoint molecules and T cell functionality, particularly in CD8+ T cells, suggesting complex immune responses by PACAP, highlighting its role in intestinal homeostasis and potential implications for inflammatory bowel diseases.

## 1. Introduction

Inflammatory bowel diseases (IBDs) are increasingly prevalent worldwide and have reached a status of global health emergency [1]. Despite extensive research, the mechanisms underlying IBDs remain unclear, and current treatments primarily manage symptoms rather than providing a cure. This study investigates the effects of PACAP deficiency on gut-associated lymphoid tissue (GALT), specifically focusing on immune cell distribution and checkpoint expression in Peyer’s patches (PPs), to enhance our understanding of immune-related bowel pathology.

The mucosa-associated lymphoid tissue (MALT) plays an important role in immunity, consisting of B-cell follicles and lymphoid aggregates [2]. In humans, GALT includes the PPs, isolated lymphoid follicles (ILF), and the appendix [3,4].

PPs are located on the antimesenteric side of the small intestine, forming clusters of lymphoid follicles. The number and size of PPs vary between species. Humans have variable number of patches, averaging 2.5 cm, whereas mice have 5–14 patches, each 3–4 mm long [3,4,5]. In humans, PPs develop prenatally, while in mice, they appear postnatally, with their numbers varying throughout life [3,4,6].

The histological structure of PPs can be divided into four regions: (1) follicle-associated epithelium (FAE); (2) subepithelial dome (SED); (3) B cell follicles, (4) and T cell zone [3,4].

PPs are linked to autoimmune diseases. In Chron’s disease, aphthoid lesions are frequently observed in the terminal ileum [7,8], while in ulcerative colitis, endoscopy shows alterations in PPs that correlate with relapse risk [9]. Gut microbiota, mediated by PPs, play a crucial role in autoimmune diseases [10]. For example, antigens of filamentous bacteria can induce follicular helper T cells in PPs, leading to autoimmune arthritis [11,12].

In recent years, immune checkpoint molecules have gained importance in cancer therapy and personalized medicine [13]. These molecules regulate immune responses through receptor-ligand interactions [14]. Key inhibitory receptors include TIM-3, PD-1, CTLA-4, and LAG-3, which exert their effects upon binding to their respective ligands: Galectin-9, PD-L1 or PD-L2, B7-1 or B7-2, and MHC class II [15]. TIM-3 is an inhibitory receptor expressed on various immune cells [16,17,18]. Its ligand, Galectin-9 (Gal-9) binds to TIM-3, inducing apoptosis in Th1 cells and promoting tolerance [19,20,21]. PD-1 is another inhibitory receptor regulating both adaptive and innate immunity [22]. Its ligands, PD-L1 and PD-L2 are expressed by various immune cells [23,24]. PD-1 interactions are important for maintaining tolerance and preventing autoimmunity [25].

Immune checkpoint molecules play an important role in maternal tolerance, transplant rejection, autoimmunity and tumor immune evasion [26]. Gut microbiota and probiotics can modulate checkpoint blockade immunotherapy through PPs [27]. PD-1 deficient mice show that PPs are affected by deregulated T-cell activity, resulting in altered IgA selection and gut dysbiosis [28].

Pituitary adenylate cyclase-activating polypeptide (PACAP) is a neuropeptide with anti-inflammatory actions acting on G protein-coupled receptors [29,30]. It plays a physiological role in the gut, regulating blood flow, motility, and secretion [31,32]. PACAP has shown beneficial effects in various gastrointestinal pathologies, including colitis [33], ileitis [34], peritonitis [35], colorectal carcinoma, and Chron’s disease [36]. It is also involved in immune homeostasis, reducing pro-inflammatory cytokine production while promoting anti-inflammatory responses [37,38].

PACAP KO mice display physiological and immunological disturbances, including altered responses in the intestines, kidneys, liver, retina, and during colitis [39,40,41,42,43,44,45,46,47,48,49,50,51,52,53,54].

This study explores immune checkpoint pathways in PPs, examining the expression of TIM-3, PD-1, Gal-9, PD-L1, and activation markers (CD69) and cytotoxicity (perforin, granzyme B) in wild-type and PACAP KO mice to better understand PACAP’s immune regulatory functions and its deficiency during aging.

## 2. Results

### 2.1. Peyer’s Patches—Macroscopic and Microscopic Features

Analyzing the mean numbers of PPs, we found qualitative differences between young and aging WT mice, the mean number of PPs was higher in WT mice at a younger age. Young and aging KO mice had similar mean numbers of PPs. Also, we found almost the same mean numbers of PPs in aging WT and KO mice (Table 1). Measuring the distances between the PPs, we did not find any regularity in any group; PPs were located randomly in the small intestine. The mean number of Peyer’s patches was significantly decreased in WT aging mice, compared to WT young mice, and in PACAP KO young mice, compared to WT young mice (Table 1).

The size of the PPs was approximately 2–4 mm in all mouse groups. In the aging group, a qualitative difference was visible between the two phenotypes; PACAP KO mice had a reduced PP thickness tendency. Histological examination of the PPs showed no difference between the groups.

### 2.2. Peyer’s Patches—Immune Cellular Features

#### 2.2.1. Phenotype Characteristics of Different T Cell Subpopulations

**Figure 1:**
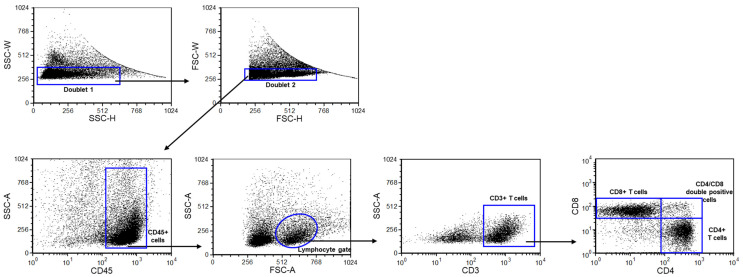
Gating strategy to identify CD3+ T, CD8+ T, CD4+, and CD4+/CD8+ T cell populations. Flow cytometric analyses for determining lymphocyte subpopulations. Following a two-step doublet exclusion, the lymphocyte population was gated using FSC-A/SSC-A parameters. From the lymphogate, CD3+ T, CD8+ T, CD4+, and CD4+/CD8+ T cell subpopulations were detected.

Based on the gating strategy, CD3+, CD8+, CD4+, and CD4+/CD8+ T lymphocyte subpopulations were determined (Figure 1), and their frequencies were compared between the investigated groups (Table 1).

Within the lymphocyte population, the percentage of CD3+ T-cells was significantly decreased in PACAP KO aging mice compared to PACAP KO young mice. The frequency of CD8+ T-cells was significantly decreased in WT aging mice compared to WT young mice. The percentage of CD4+ T-cells significantly decreased in PACAP KO aging mice compared to WT aging mice.

Within the CD3+ T-cells, the frequency of CD8+ T-cells was significantly decreased in WT aging mice compared to WT young mice. The frequency of CD4+ T-cells ratio in CD3+ T-cells was significantly decreased in PACAP KO aging mice, compared to WT aging mice. Furthermore, we found a significant increase in aging WT mice, compared to young WT mice regarding the frequency of CD4+ T-cells in CD3+ T-cells.

The frequency of CD8+ T-cells in CD3+ T-cells was significantly decreased in PACAP KO aging mice, compared to PACAP KO young mice; furthermore, it was significantly higher in PACAP KO young mice, compared to WT young mice (Table 1).

#### 2.2.2. Immune Checkpoint Molecules

##### PD-1 and PD-L1 Expression by Different T Cell Subpopulations

The PD-1 expression by CD3+ and CD4+ T-cells was significantly decreased in PACAP KO aging mice, compared to WT aging mice and significantly decreased in PACAP KO aging mice, compared to PACAP KO young mice. The PD-1 expression by CD8+ T-cells significantly decreased in PACAP KO aging mice compared to PACAP KO young mice (Table 1).

**Figure 2:**
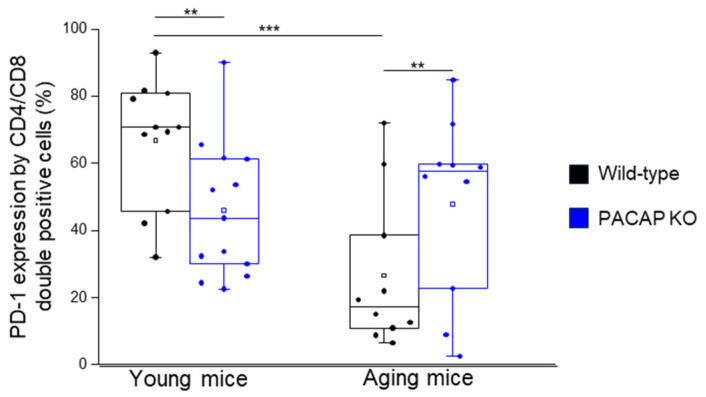
PD-1 expression by CD4+/CD8+ T-cells in young and aging WT and PACAP KO mice. The surface expression of PD-1 receptor by CD4+/CD8+ T-cells in young and aging WT and PACAP KO mice. The solid bars represent medians of 11, 13, 10, and 10 determinations, respectively. The boxes indicate the interquartile ranges, and the whiskers represent the variability of the minimum, maximum, and any outlier data points in comparison to the interquartile range. The middle square within the box represents the mean value. Significant differences with *p*-values < 0.05 ** < 0.001 *** are indicated.

PD-1 expression by CD4+/CD8+ T-cells was significantly decreased in PACAP KO young mice, compared to WT young mice, while in PACAP KO aging mice, we found a significant increase, compared to the WT aging mice. PD-1 expression by CD4+/CD8+ T-cells was significantly decreased in WT aging mice compared to WT young mice (Figure 2).

The PD-L1 expression by all three immune cell populations (CD3+, CD4+, and CD8+ T-cells) was significantly decreased in PACAP KO aging mice, compared to PACAP KO young mice, while PD-L1 expression by CD4+/CD8+ T-cells did not show any significant difference between the different mouse groups (Table 1).

##### TIM-3 and Galectin-9 Expression by Different T Cell Subpopulations

**Figure 3:**
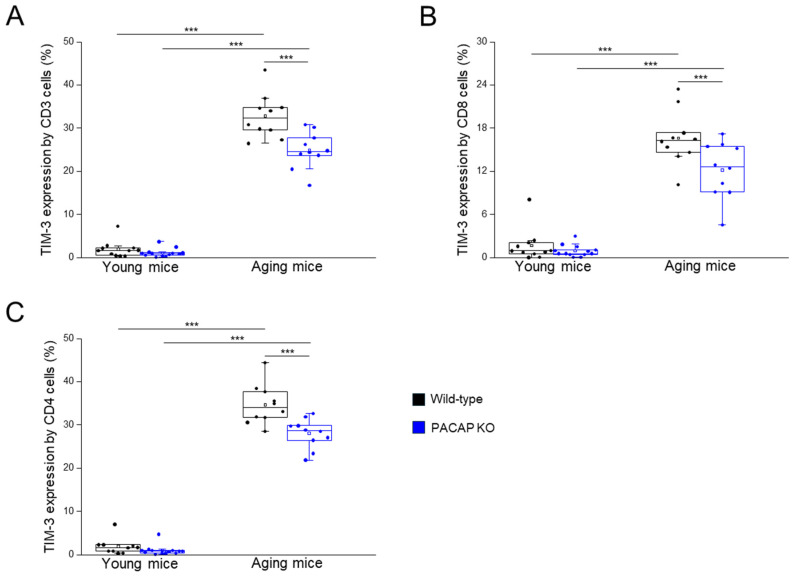
TIM-3 expression by CD3+, CD4+, and CD8+ T-cells in young and aging WT and PACAP KO mice. The surface expression of TIM-3 receptor by CD3+ (**A**), CD8+ T (**B**), and CD4+ (**C**) cells in young and aging WT and PACAP KO mice. The solid bars represent medians of 10, 10, 10, and 10 determinations, respectively. The boxes indicate the interquartile ranges, and the whiskers represent the variability of the minimum, maximum, and any outlier data points in comparison to the interquartile range. The middle square within the box represents the mean value. Significant differences with *p*-values < 0.001 *** are indicated.

The TIM-3 expression by all investigated immune cell populations (CD3+, CD4+, CD8+, and CD4+/CD8+ T-cells) significantly decreased in PACAP KO aging mice, compared to WT aging mice, significantly higher in WT aging mice, compared to WT young mice, and significantly higher in PACAP KO aging mice, compared to PACAP KO young mice (Figure 3A–C and Table 1).

Gal-9 expression by CD3+, CD4+, CD8+, and CD4+/CD8+ T-cells was significantly decreased in WT aging mice, compared to WT young mice, and significantly decreased in PACAP KO aging mice, compared to PACAP KO young mice (Table 1).

#### 2.2.3. Expression of Intracellular Molecules by Different T Cell Subpopulations

The perforin expression by CD3+ and CD8+ T-cells was significantly higher in PACAP KO young mice compared to WT young mice.

**Figure 4:**
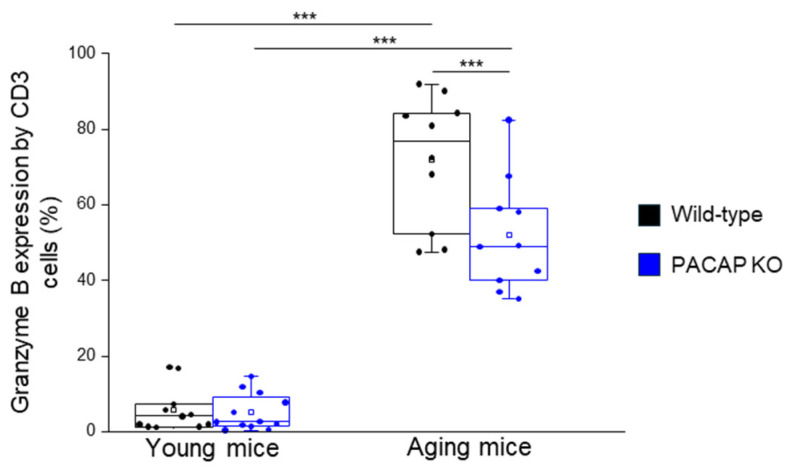
Granzyme B expression by CD3+ T-cells in young and aging WT and PACAP KO mice. The intracellular expression of Granzyme B by CD3+ T-cells in young and aging WT and PACAP KO mice. The solid bars represent medians of 10, 9, 10, and 5 determinations, respectively. The boxes indicate the interquartile ranges, and the whiskers represent the variability of the minimum, maximum, and any outlier data points in comparison to the interquartile range. The middle square within the box represents the mean value. Significant differences with *p*-values < 0.001 *** are indicated.

The granzyme B expression by CD3+ T-cells was significantly decreased in PACAP KO aging mice, compared to WT aging mice, significantly higher in WT aging mice, compared to WT young mice, and significantly higher in PACAP KO aging mice, compared to PACAP KO young mice. The granzyme B expression by CD8+ T-cells was significantly higher in WT aging mice, compared to WT young mice, and PACAP KO aging mice, compared to PACAP KO young mice (Figure 4 and Table 1).

#### 2.2.4. CD69 Expression by Different T Cell Subpopulations

**Figure 5:**
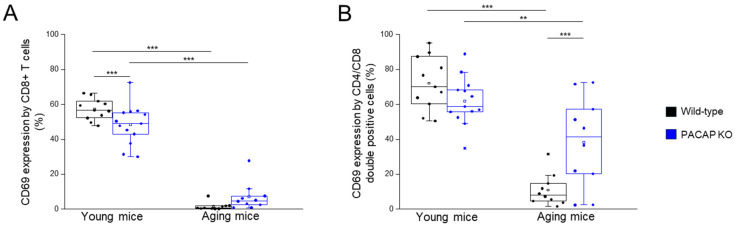
CD69 expression by CD8+ and CD4+/CD8+ T cells in young and aging WT and PACAP KO mice. The surface expression of CD69 receptor by CD8+ (**A**) and CD4+/CD8+ (**B**) T cells in young and aging WT and PACAP KO mice. The solid bars represent medians of 10, 10, 10, and 10 determinations, respectively. The boxes indicate the interquartile ranges, and the whiskers represent the variability of the minimum, maximum, and any outlier data points in comparison to the interquartile range. The middle square within the box represents the mean value. Significant differences with *p*-values < 0.01 ** < 0.001 *** are indicated.

The CD69 expression by all investigated immune cells (CD3+, CD4+, and CD8+ T-cells) was significantly decreased in WT aging mice, compared to WT young mice, and significantly decreased in PACAP KO aging mice, compared to PACAP KO young mice (Figure 5A,B and Table 1).

The CD69 expression by CD4+/CD8+ T-cells was significantly higher in PACAP KO aging mice, compared to WT aging mice, significantly decreased in WT aging mice, compared to WT young mice, and significantly decreased in PACAP KO aging mice, compared to PACAP KO young mice (Figure 5B and Table 1).

## 3. Discussion

PACAP levels decline with age, correlating with increased tissue vulnerability and various age-related abnormalities such as retinal degeneration, altered lipid metabolism, accelerated aging, and increased mortality [39,42,55]. Our study highlights PACAP’s role in aging and the need for further research to validate these findings and explore therapeutic interventions targeting PACAP.

PACAP is critical for maintaining tissue integrity and may influence inflammatory responses and degenerative diseases [56,57,58,59,60,61]. Elevated PACAP levels in ulcerative colitis patients suggests its role in IBDs, emphasizing its importance in intestinal homeostasis [62]. Studies show PACAP KO mice are more susceptible to colitis and colorectal cancer, underscoring PACAP’s protective role against severe colonic inflammation and carcinogenesis [36,54]. Altered microbiota composition, including a significant reduction in Bifidobacteria, contributes to this vulnerability [63].

Macroscopic features consistent with previous studies identified in humas, with random arrangement and variable distances between PPs along the small intestine [5]. Confirming previous reports, the size of the mouse PPs was between 2–4 mm [3,4]. The number of PP peaks in adolescence [4,5], and was higher in the young (3 months old) WT animals than in the aging (12–15 months) WT animals, a difference not observed in PACAP KO mice. Earlier studies showed amyloid depositions in PACAP-deficient tissues during age-related senile systemic amyloidosis [39,55]. Our histological analysis of PPs revealed no differences between young WT and KO mice. However, significant alterations in immune cell populations and immune checkpoint molecule expression were observed in aging PACAP KO mice compared to WT mice, suggesting altered immune regulation due to PACAP deficiency.

Aging impacts oral tolerance through the decline of Peyer’s patch functionality [64]. Elevated levels of PACAP38-positive cells have been documented in peripheral lymphoid tissues and in the duodenal mucosa [65]. PACAP immunoreactivity in neurons and fibers of ENS and the GALT [66], along with VPAC2 receptor presence in PPs [67], suggests a close regulatory connection between the immune and neuroendocrine systems in maintaining intestine homeostasis. The immune cellular findings in KO mice PPs could be attributed to the lack of PACAP.

Flow cytometric analysis revealed that aging in WT mice leads to a significant reduction in CD8+ T cells, similar to findings in elderly humans [68,69]. Aging CD8+ T cells showed higher TIM-3 expression and reduced Gal-9 expression, along with decreased CD69 expression, indicating reduced activation [70,71,72]. Despite unchanged perforin levels, aging CD8+ T cells exhibited upregulated granzyme B expression.

In contrast, aging WT mice showed a higher frequency of CD4+ T cells and a progressive increase in CD4+ T cells that lack CD28 expression, similar to aging in healthy humans [73,74,75]. Aging CD4+ T cells displayed higher TIM-3 expression and reduced Gal-9 expression, suggesting regulatory functions. Changes in PD-1 expression in aging WT mice were consistent with decreased T cell functional capacity.

Aging PACAP KO mice showed decreased PD-1, PD-L1, Gal-9, and CD69 expression in CD3+/CD4+ and CD3+/CD8+ T cells, with significantly higher TIM-3 expression, similar to findings in aging WT mice. Functionally, aging CD8+ T cells in PACAP KO mice exhibited higher granzyme B expression [76]. Contrary to common assumptions, clonal expansion of CD8+ T cells, rather than cytotoxic activity, is the decisive factor in the observed reduced CTL response in aging mice [77].

After examining the effects of aging on the immune system in both groups of mice, we evaluated differences between young groups (young WT vs. young PACAP KO) and how these evolved with age (aging WT vs. aging PACAP KO).

Investigating differences between young WT and PACAP KO mice, we found no significant differences in overall immune cell population frequencies between the two young groups.

Despite a significant decrease in PD-1 expression by the CD4+/CD8+ T cell population in young PACAP KO mice compared to young WT mice, functional analysis revealed reduced activation in young PACAP KO mice. This reduction occurred alongside a significant increase in perforin expression in both CD3+ and CD8+ T cells in young PACAP KO mice compared to WT mice. These findings suggest that altered checkpoint molecule expression does not necessarily translate into enhanced T-cell activation.

Our investigation revealed key differences in immune cell profiles and checkpoint molecule expression between aging PACAP KO and aging WT mice. Specifically, aging PACAP KO mice showed a significant decrease in CD4+ T cells compared to WT mice, accompanied by decreases in PD-1 and TIM-3 expression, suggesting modulation of immune tolerance mechanisms in the absence of PACAP. However, the functional parameters of these cells remained similar to those observed in aging WT mice.

In aging PACAP KO mice, CD8+ T cells showed significantly reduced TIM-3 expression compared to aging WT mice. The CD4+/CD8+ T-cell population in aging PACAP KO mice demonstrated increased activity, correlating with the decreased TIM-3 expression. These results suggest a compensatory mechanism that may enhance T-cell activity in the absence of PACAP to maintain immune responsiveness.

The total CD3+ T-cell population in aging PACAP KO mice exhibited a significant reduction in granzyme B expression despite decreases in PD-1 and TIM-3 inhibitory checkpoint molecules. This interplay of changes underscores the nuanced impact of PACAP deficiency on immune regulation, particularly in the context of aging.

These findings underscore the critical role of PACAP in maintaining immune homeostasis. Our study illuminates PACAP’s potential role in maintaining immune homeostasis within Peyer’s patches and its significant implications for IBDs and aging. Dysregulation of immune cell populations and checkpoint molecules in PACAP KO mice underscores PACAP essential role in modulating immune responses. This dysregulation, coupled with elevated PACAP expression observed in ulcerative colitis patients, suggests that PACAP deficiency could exacerbate IBDs by disrupting mucosal immunity [62].

Furthermore, the age-related decline in immune function, characterized by altered T cell profiles in aging PACAP KO mice, underscores the importance of PACAP in preserving immune integrity with age. These insights position PACAP as a potential therapeutic target for mitigating chronic inflammation and enhancing immune function in both IBDs and the aging population, potentially leading to novel treatment strategies that could improve health outcomes.

Nevertheless, our study also bears several limitations. First, the number of immune cells from the Peyer’s patches available for experiments was limited, thus restricting the range of experiments we could perform, including further phenotypical and functional assays, which could have added substantial value to the study. Second, we focused primarily on T cell populations and did not explore other immune cells such as B cells or macrophages, which could provide a more comprehensive view. Third, the use of 12–15-month-old mice represents early immune dysfunction rather than full immunosenescence, and older mice would better model advanced aging. Furthermore, while we examined Peyer’s patches, the findings may not extend to other immune tissues. Finally, using a single animal model without human data validation limits the generalizability of our results.

In conclusion, our study underscores the importance of PACAP in maintaining immune homeostasis in gut-associated lymphoid tissue. The absence of PACAP leads to significant alterations in immune cell populations, particularly evident with aging. These findings enhance our understanding of PACAP’s integral role in gut immunity and its potential implications for inflammatory bowel diseases.

## 4. Materials and Methods

### 4.1. Animal Model

We used PACAP-deficient mice generated on a CD-1 background, as previously described [78]. The animals were divided into four groups: young wild-type (WT) (3-month-old, *n* = 10), young PACAP knockout (PACAP KO) (3-month-old, *n* = 14), aging WT (12–15-month-old, *n* = 10) and aging PACAP KO (12–15-month-old, *n* = 18) male mice. They were maintained on a 12 h light/dark cycle at 20–22 °C and 40–60% humidity and were fed with standard feed pellets and tap water. Animals were handled in accordance with an approved protocol by the animal ethics committee of the University of Pecs (BA/73/00452-6/2023). Genotyping was performed using Phire Animal Tissue Direct PCR Kit (Thermo Fisher Scientific, Waltham, MA, USA) according to the manufacturer’s instructions. Primer sequences used to detect wild-type and KO DNA signatures of PACAP were identical to those used earlier [41,78].

### 4.2. Isolation of the Peyer’s Patches from the Small Intestine

Both young and aging groups of WT and PACAP KO male mice (*n* = 10 in each group) were euthanized with intraperitoneal sodium pentobarbital at a dose of 100 mg/kg. After the anesthesia, we opened the abdominal cavity and removed the gastrointestinal tract. We counted the number of Peyer’s patches and measured the length of the small intestine, the size of the PPs, and the distance between the neighboring PPs. We removed the stomach and the large intestine, and then, with the help of a syringe, we washed out the faces with PBS from the lumen of the small intestine, then we isolated the PPs [79].

### 4.3. Histological Analysis

For histological analysis, one PP from each mouse was fixed in 4% buffered paraformaldehyde, embedded in paraffin, sectioned at 3 μm, and stained with hematoxylin and eosin. (Paraform Sectionable Cassette System, Tissue-Tek X-Press, AutoTek120, and Prisma Film Coverslipper HQ smart automation system, Sakura Finetek, Alphen aan den Rijn, The Netherlands). Later, we performed a qualitative analysis of the slides, analyzing the number, the size, and the distribution of the PPs.

### 4.4. Cell Isolation from the Peyer’s Patches

We employed mechanical disaggregation to isolate immune cells from PP tissues, minimizing selective cell loss and preserving surface protein integrity. Later, PPs were homogenized thoroughly with a syringe plunger, and single-cell suspensions were prepared using a 100 μm nylon cell strainer (BD Biosciences, Franklin Lakes, NJ, USA). Subsequently, cells were washed in PBS and the supernatant was aspirated. The pellet was resuspended in PBS and filtered again via a 70 μm nylon cell strainer (BD Biosciences, Franklin Lakes, NJ, USA). Then, cells were washed in PBS, and the supernatant was aspirated and filtered via a 40 μm nylon cell strainer (BD Biosciences, Franklin Lakes, NJ, USA). Cells were washed again in PBS, and the supernatant was aspirated.

### 4.5. Mononuclear Cell Surface Staining, Antibodies, and Flow Cytometric Analysis

Isolated cells were suspended in PBS and incubated with fluorochrome-labeled monoclonal antibodies at room temperature for 30 min for surface characterization. Antibodies used in the present study are shown in Table 2. The samples were protected from light. After washing, the cells were resuspended in 300 µL PBS containing 1% paraformaldehyde and stored at 4 °C in complete darkness until fluorescence-activated cell sorting (FACS) analysis. Before sample analysis, the settings of the flow cytometer were checked using Cytometer Setup and Tracking beads (CS&T beads; BD Biosciences, Franklin Lakes, NJ, USA) according to the manufacturer’s instructions. Compensation beads were used with single stains of each antibody to determine the compensation settings and were applied in FACS Diva V6 software (BD Biosciences, Franklin Lakes, NJ, USA, Version 6, accession date: 1 September 2023) before data collection. Labeled cells were analyzed with FACS Canto II flow cytometer (BD Biosciences, Franklin Lakes, NJ, USA), and 10,000 events were collected in the lymphogate after CD45 staining. FACS Diva V6 software was used for data acquisition and FCS Express IV software (De Novo Software, version 4, accession date: 1 September 2023) for data analysis.

### 4.6. Intracellular Staining of Perforin and Granzyme B

After surface labeling, cells were washed and fixed in 4% PFA for 10 min at room temperature. Next, the cells were washed with PBS and incubated with 1:10 diluted FACS Permeabilizing Solution 2 (BD Biosciences, Franklin Lakes, NJ, USA) for 10 min at room temperature. The cells were then incubated with anti-mouse granzyme B and anti-mouse perforin for 30 min at room temperature in complete darkness. The samples were washed with PBS, fixed with 1% PFA, and stored at 4 °C in the dark until FACS analysis.

### 4.7. Statistical Analysis

Statistical analyses were performed using the SPSS version 28.0 (IBM, New York, NY, USA). Two-way ANOVA with multiple pairwise comparisons was used to determine the effect of age and group on interest in immunological parameters, such as the number of Peyer’s patches. A partial eta squared value was calculated for statistically significant findings to demonstrate the effect size. Effect sizes for the two-way ANOVA test were indicated as small where the η^2^ was between 0.01 and 0.06, moderate where η^2^ was between 0.06 and 0.14, and large effect where the rank η^2^ was greater than 0.14. Pearson product-moment correlation was used to define the linear correlation between immunological parameters. Pearson’s correlation coefficient scale indicates 0.00–0.19 = very low, 0.20–0.39 = low, 0.40–0.59 = moderate, 0.60–0.79 = high, and 0.80–1.00 = very high correlation. The *p*-values less than 0.05 were considered to be significant.

## Figures and Tables

**Table 1:** Phenotypic and functional characteristics of immune cells. The results are presented as the mean value ± SD. Statistical comparisons were made using two-way ANOVA tests. Differences were considered statistically significant for *p*-values ≤ 0.05, NS: not significant.

	WT Young	PACAP KO Young	WT Aging	PACAP KO Aging	*p* Value
CD3+ T-cells	50.38 ± 13.52	53.24 ± 17.71	42.18 ± 13.39	37.76 ± 9.68	young KO vs. aging KO *p* = 0.013
CD8+ T-cells	15.33 ± 10.40	14.23 ± 9.93	5.03 ± 2.27	8.57 ± 5.68	young WT vs. aging WT *p* = 0.006
CD4+ T-cells	28.67 ± 11.63	27.40 ± 10.88	32.64 ± 11.21	20.67 ± 5.30	aging WT vs. aging KO *p* = 0.012
CD8+ T-cells ratio in CD3+ T-cells	28.74 ± 17.70	26.94 ± 14.62	12.20 ± 6.22	21.44 ± 9.23	young WT vs. aging WT *p* = 0.006
CD4+ T-cells ratio in CD3+ T-cells	59.27 ± 23.33	53.83 ± 19.79	77.19 ± 9.96	55.70 ± 11.07	aging WT vs. aging KO *p* = 0.009 young WT vs. aging WT *p* = 0.024
CD4+ T-cells ratio in CD8+ T-cells	3.75 ± 5.10	8.63 ± 8.13	1.12 ± 1.11	3.40 ± 2.33	young KO vs. aging KO *p* = 0.023 young WT vs. Young KO *p* = 0.029
PD-1 expression by CD3+ T-cells	32.18 ± 11.64	31.48 ± 13.58	30.27 ± 9.80	19.88 ± 7.75	aging WT vs. aging KO *p* = 0.043 young KO vs. aging KO *p* = 0.018
PD-1 expression by CD4+ T-cells	36.53 ± 11.49	32.85 ± 11.46	33.32 ± 8.72	21.08 ± 5.65	aging WT vs. aging KO *p* = 0.008 young KO vs. aging KO *p* = 0.007
TIM-3 expression by CD4+/CD8+ T-cells	7.07 ± 6.96	2.36 ± 1.48	30.94 ± 8.33	22.01 ± 4.32	aging WT vs. aging KO *p* = 0.001 young WT vs. aging WT *p* < 0.001 young KO vs. aging KO *p* < 0.001
CD69 expression by CD3+ T-cells	41.26 ± 10.23	38.47 ± 12.94	7.53 ± 1.52	9.46 ± 6.54	young WT vs. aging WT *p* < 0.001 young KO vs. aging KO *p* < 0.001
CD69 expression by CD4+ T-cells	30.61 ± 5.31	27.92 ± 11.09	6.00 ± 1.49	4.11 ± 2.77	young WT vs. aging WT *p* < 0.001 young KO vs. aging KO *p* < 0.001
Gal-9 expression by CD3+ T-cells	46.12 ± 17.70	55.44 ± 13.68	10.32 ± 8.12	14.97 ± 10.06	young WT vs. aging WT *p* < 0.001 young KO vs. aging KO *p* < 0.001
Gal-9 expression by CD8+ T-cells	52.40 ± 15.63	59.17 ± 12.08	12.54 ± 9.07	13.71 ± 8.05	young WT vs. aging WT *p* < 0.001 young KO vs. aging KO *p* < 0.001
Gal-9 expression by CD4+ T-cells	40.05 ± 13.98	50.20 ± 17.24	7.60 ± 6.84	10.94 ± 5.90	young WT vs. aging WT *p* < 0.001 young KO vs. aging KO *p* < 0.001
Gal-9 expression by CD4+/CD8+ T-cells	78.59 ± 12.11	73.70 ± 16.55	29.08 ± 21.25	44.15 ± 22.00	young WT vs. aging WT *p* < 0.001 young KO vs. aging KO *p* < 0.001
PD-L1 expression by CD3+ T-cells	48.26 ± 30.09	57.54 ± 23.75	35.52 ± 10.32	30.51 ± 8.38	young KO vs. aging KO *p* = 0.004
PD-L1 expression by CD4+ T-cells	49.14 ± 30.57	58.81 ± 23.82	30.81 ± 11.38	23.89 ± 9.69	young KO vs. aging KO *p* < 0.001
PD-L1 expression by CD8+ T-cells	46.53 ± 28.39	55.28 ± 23.80	35.23 ± 10.38	31.44 ± 8.19	young KO vs. aging KO *p* = 0.009
PD-L1 expression by CD4+/CD8+ T-cells	63.53 ± 33.92	69.05 ± 23.98	53.40 ± 12.19	53.87 ± 14.07	NS
perforin expression by CD3+ T-cells	42.41 ± 24.32	60.93 ± 18.97	43.54 ± 8.21	49.57 ± 21.71	young WT vs. Young KO *p* = 0.026
perforin expression by CD8+ T-cells	51.12 ± 27.26	67.91 ± 16.65	47.84 ± 8.32	55.35 ± 23.29	young WT vs. Young KO *p* = 0.05
Granzyme B expression by CD8+ T-cells	8.58 ± 7.72	7.61 ± 5.02	74.39 ± 14.67	71.13 ± 15.99	young WT vs. aging WT *p* < 0.001 young KO vs. aging KO *p* < 0.001
Number of Peyer’s patches (PP)	12.40 ± 2.72	10.46 ± 1.69	10.20 ± 1.69	10.11 ± 1.81	young WT vs. aging WT *p* = 0.016 young WT vs. Young KO *p* = 0.015

**Table 2:** Fluorochrome conjugated monoclonal antibodies used in the study.

Antigen	Format	Clone	Isotype	Company	CAT
CD3	BV510	145-2C11	Armenian Hamster IgG1, κ	BD Biosciences	563024
CD4	FITC	GK1.5	Lewis IgG2b, κ	BD Biosciences	557307
CD8	APC-H7	53-6.7	Louvain, LOU/C, LOU/M IgG2a, κ	BD Biosciences	560247
CD45	PerCp	30-F11	Louvain, LOU/C, LOU/M IgG2b, κ	BD Biosciences	561047
CD69	PE-Cy7	H1.2F3	Armenian Hamster IgG1, λ3	BD Biosciences	552879
Galectin-9	BV421	RG9-35	Rat IgG2a, κ	BD Biosciences	566028
GranzymeB	FITC	REA226	recombinant human IgG1	Miltenyi Biotec.	130-118-341
PD-1	BV421	J43	Armenian Hamster IgG2, κ	BD Biosciences	562584
PD-L1	APC	MIH5	Sprague-Dawley (outbred) IgG2a, λ	BD Biosciences	564715
Perforin	APC	S16009A	Rat IgG2a, κ	Biolegend	154304
TIM-3	APC	215008	Rat IgG2A	R&D Systems	FAB1529A

## Data Availability

Data are contained within the article.
